# DDX RNA helicases: key players in cellular homeostasis and innate antiviral immunity

**DOI:** 10.1128/jvi.00040-24

**Published:** 2024-08-30

**Authors:** Iulia Tapescu, Sara Cherry

**Affiliations:** 1 Department of Pathology and Laboratory Medicine, University of Pennsylvania, Philadelphia, Pennsylvania, USA; 2 Biochemistry and Biophysics Graduate Group, University of Pennsylvania, Philadelphia, Pennsylvania, USA; Indiana University Bloomington, Bloomington, Indiana, USA

**Keywords:** antiviral, helicase, RNA-binding protein, innate immunity, replication, DEAD, DDX, host factor

## Abstract

RNA helicases are integral in RNA metabolism, performing important roles in cellular homeostasis and stress responses. In particular, the DExD/H-box (DDX) helicase family possesses a conserved catalytic core that binds structural features rather than specific sequences in RNA targets. DDXs have critical roles in all aspects of RNA metabolism including ribosome biogenesis, translation, RNA export, and RNA stability. Importantly, functional specialization within this family arises from divergent N and C termini and is driven at least in part by gene duplications with 18 of the 42 human helicases having paralogs. In addition to their key roles in the homeostatic control of cellular RNA, these factors have critical roles in RNA virus infection. The canonical RIG-I-like receptors (RLRs) play pivotal roles in cytoplasmic sensing of viral RNA structures, inducing antiviral gene expression. Additional RNA helicases function as viral sensors or regulators, further diversifying the innate immune defense arsenal. Moreover, some of these helicases have been coopted by viruses to facilitate their replication. Altogether, DDX helicases exhibit functional specificity, playing intricate roles in RNA metabolism and host defense. This review will discuss the mechanisms by which these RNA helicases recognize diverse RNA structures in cellular and viral RNAs, and how this impacts RNA processing and innate immune responses.

## RNA HELICASES: STRUCTURAL DIVERSITY AND REGULATORY FUNCTION

RNAs have diverse regulatory roles beyond just carriers of genetic information (1). RNA biogenesis includes numerous post-transcriptional modifications and processing steps that influence RNA stability, folding, and interactions with cellular factors (2). RNAs adopt structures enabling interactions with RNA-binding proteins (RBP) and other RNA molecules, serving as pivotal regulators (3). RNAs are flexible, forming various stable structures including hairpins, G-quadruplexes, Z-RNA, pseudoknots, double strands, R-loops, and others (4). These structures influence many cellular processes including mRNA stability and localization, ribosomal biogenesis, splicing, and translation (5–11). In addition, RNA viruses encode many RNA secondary structures in their compact genomes, directing diverse aspects of their life cycles (12–21). To recognize viral RNAs as foreign invaders, cellular RNA binding proteins bind to specialized features to restrict replication.

**Fig 1:**
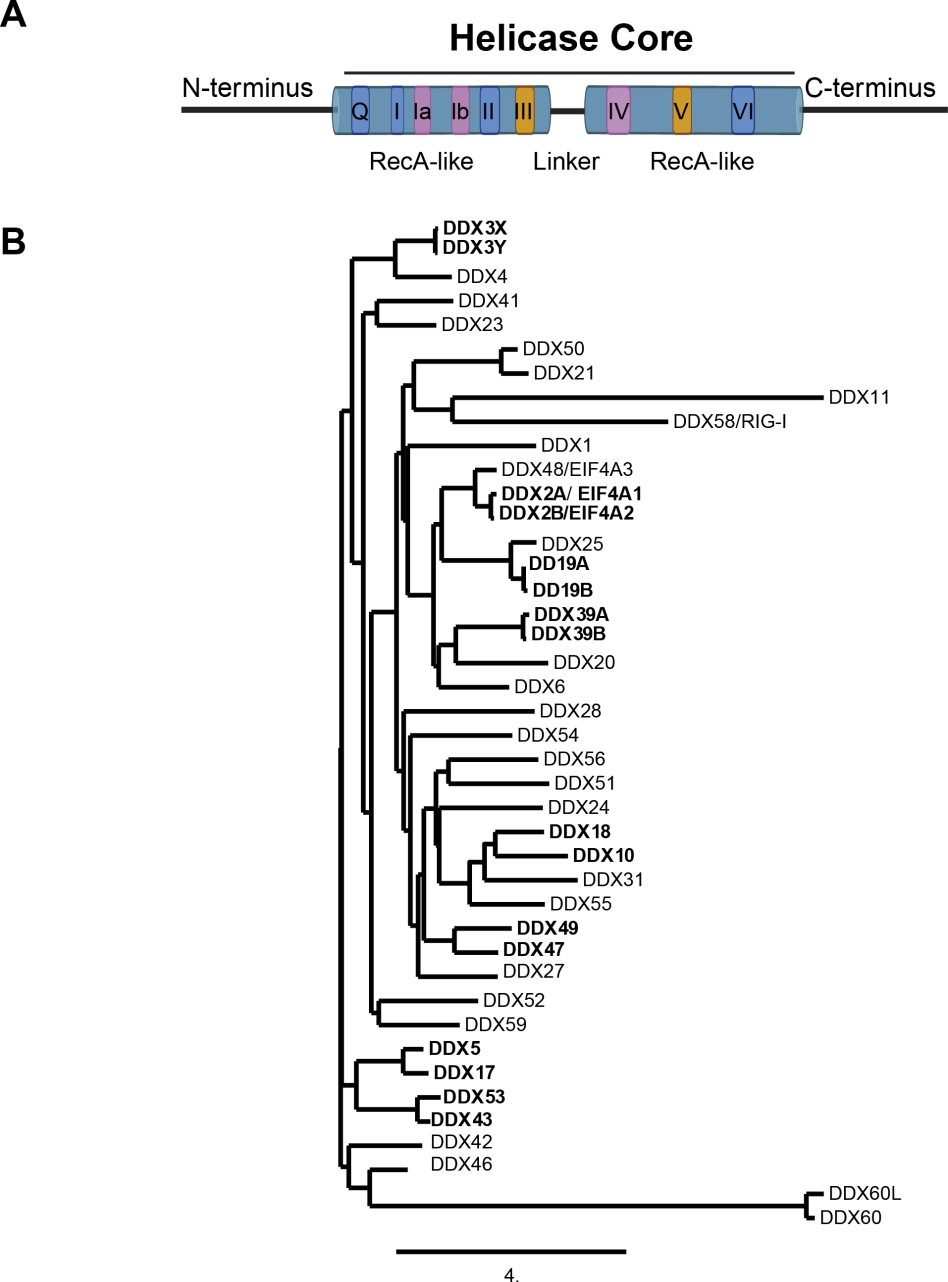
The general structure of DExD helicases and phylogenetic tree. (**A**) The helicase core is composed of two RecA-like domains connected by a flexible linker and divergent N- and C-termini, which provide RNA and/or protein interaction specificity. Motifs Q, I, II, and VI regulate ATP binding and hydrolysis (blue), motifs Ia, Ib, and IV facilitate RNA binding (purple), and motifs III and V coordinate ATP hydrolysis and RNA unwinding (orange). The figure was not drawn to scale. (**B**) The phylogenetic tree of 42 human DDX with paralogs bolded was generated with http://www.phylogeny.fr (45–50) using protein sequences of canonical isoforms (Table S1).

The binding of many RNA structures is mediated by RNA helicases, first identified in the 1980s (22). RNA helicases fall into six superfamilies (23). DExD/H (DDX/H) box helicases belonging to the superfamily 2 form the largest family of RNA helicases and are present in all domains of life (24, 25). While many DDX helicases interact with RNA substrates along the sugar-phosphate in a sequence-non-specific manner, they exhibit functional specificity. Current literature highlights the broad yet specific roles of DDX helicases in RNA metabolism within cellular function and host defense. Structurally, DDXs possess divergent N- and C-termini that flank a relatively conserved helicase core, comprising two RecA-like domains connected by a flexible linker region (26–29) (Fig. 1A). The helicase core features several conserved motifs, including motif II (D-E-x-D, Aspartate-Glutamate-X-Aspartate), from which the family derives its name, along with other motifs crucial for RNA and ATP binding and hydrolysis (30–37). Apart from unwinding RNA duplexes, DDXs perform various functions such as strand annealing, G4-quadruplex unwinding (38–40), protein-displacement (41, 42), and recognition of secondary structures on viral RNAs (43, 44).

Mechanistically, RNA helicases behave as molecular switches adopting different conformations in the presence or absence of nucleotide and RNA substrates. In the absence of ATP or RNA substrates, DExD-box helicases are in an open conformation. Upon binding to both ATP and RNA, the open conformation switches to an active closed conformation (33). The closed helicase conformation can lead to RNA duplex unwinding and release and ATP hydrolysis, which are thought to be required for the recycling of the helicase protein (51, 52). Unlike other helicases, DDX proteins can unwind duplexes in either a 5′ or 3′ direction and can even unwind blunt-ended RNAs without needing a specific polarity, suggesting that DDXs can alter internal RNA structures (53–55). The helicase core of DDX proteins interacts with the sugar-phosphate backbone of the RNA substrates thus binding to the RNA substrate is broadly structure dependent but sequence independent (55, 56). Therefore, while DDXs use conserved motifs to interact with their substrates similarly, sequence divergence among helicases drives the specificity for distinct RNA targets (57). For example, the RNA-binding cleft is positively charged in all DDXs, but the size of the patch differs, which may accommodate different RNA structures (57). There is also a variable loop within the helicase core and a flexible linker that clamps RNA ligands in specific conformations that may alter the enzymatic efficiency of DDX helicases (57).

## MECHANISTIC INSIGHTS AND FUNCTIONAL DIVERSITY OF RNA HELICASES

Biochemically, DDX/H proteins can unwind duplexes through RNA-dependent ATPase and ATP-dependent activities. In addition, these RNA helicases generally exhibit low processivity and unwind relatively short 10–25 bp duplex RNAs (58–60). For example, DDX2 (elF4A), the prototypical DDX, unwinds 10–15 bp duplex RNA (53), and similarly, DDX39A and DDX39B unwind 10 bp and 13 bp duplex RNAs, respectively (58, 59). Since helical elements within structured RNA rarely exceed 10 bp, the activities of DDXs to unwind short local structures are sufficient for their endogenous function (61).

These activities can be modulated by protein-binding partners or specific RNA ligands. For example, the ATPase activity of DDX46 is more efficient in the presence of its target U2 snRNA compared to other snRNAs (58, 62), while DDX49’s ATPase is inhibited by RNA (63). Broadly, some DDXs show more efficient ATPase activity with single-stranded substrates, while others prefer substrates with double-stranded character (58, 64). However, not much is known about the mechanism underlying these differences. Protein-protein interactions also impact function. DDX6 interactions with proteins MIF4G and CNOT1 boost its ATPase activity (65), as does a specific viral RNA target (66). On the other hand, the relatively weak ATPase and helicase activities of most DDX suggest that other protein factors are needed to stimulate these functions. For instance, CIP29 stimulates the DDX39A helicase activity *in vitro*, but the spectrum of binding partners that alter the biochemical properties of DDXs is poorly understood (59). In addition to unwinding activity, DDXs also can remodel RNA-protein complexes. For example, DDX3X remodels the cap-binding complex and eIF3 on uORF-containing mRNAs to enhance translation (67). DDXs can also act as scaffolds to recruit or alter protein-protein interactions using auxiliary domains or unstructured regions (68, 69). Furthermore, they can be regulated in diverse ways including localization, and post-translational modifications. For instance, the localization of these helicases in various compartments, such as the nucleolus, alters the spectrum of binding partners (70). PTMs from phosphorylation to ubiquitylation have been observed for many helicases, and notably, although posttranslational regulation occurs throughout, the N- and C-termini are favored sites for regulation, involving both PTMs and protein-protein interactions (71). However, the mechanisms by which these modifications alter the biochemical properties, and the biological functions of the helicases are only understood for a handful of helicases (71–74). The best studied is DDX58, where K63-linked polyubiquitination induces oligomerization and antiviral gene expression (75). Additional PTMs such as phosphorylation acetylation, SUMOylation, and ISGylation further regulate DDX58 transition between activated and inactivated states (75). Pathogenic point mutations in DDX58, which lead to aberrant ubiquitination have also been found in patients with autoimmune diseases such as lupus nephritis (76). Likewise, localization can be regulated by PTMs adding another level of complexity to the functions and specificity of these RNA-binding proteins (59, 77–83).

## EVOLUTIONARY EXPANSION AND FUNCTIONAL DIVERSIFICATION

**Fig 2:**
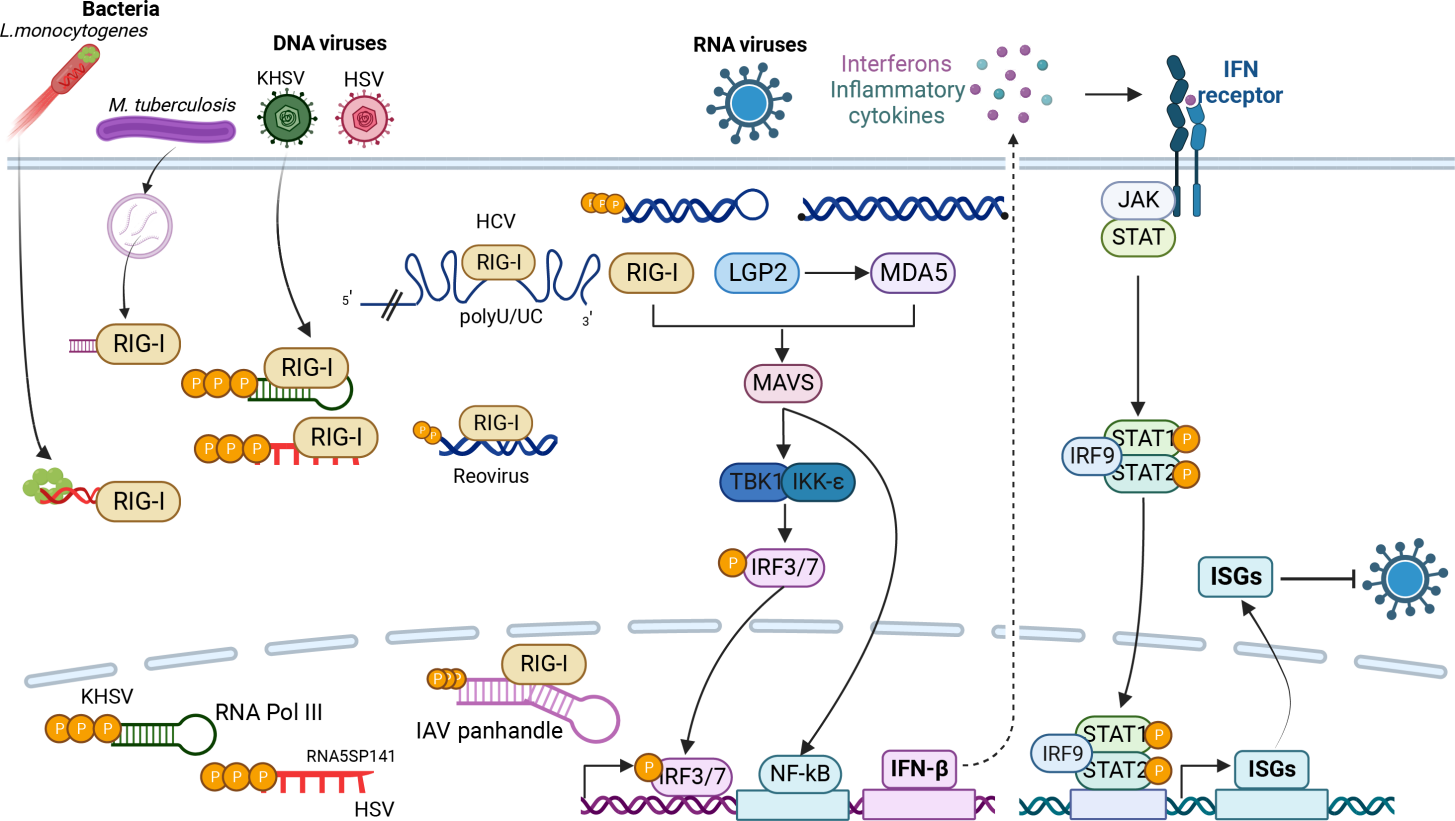
RLR activation by diverse pattern recognition motifs leads to interferon (IFN) activation and antiviral gene expression. RIG-I binds to 5′triphosphates on short dsRNA substrates but also senses a 5′diphosphate on reovirus, polyU/UC motifs on HCV 3′ UTR, and an IAV genomic panhandle in the nucleus. In addition to sensing RNA viruses, RIG-I can else detect host 5′triphosphate mRNAs synthesized by RNA pol III from DNA genomes of DNA viruses including HSV and KHSV. The RLR pathway senses RNA complexes with bacterial RNA from *L. monocytogenes* and *M. tuberculosis* RNA. LGP2 enhances the activity of MDA-5, which binds to long dsRNA substrates. RLR binding to viral RNAs promotes MAVS binding and activation of the TANK-binding kinase 1/IκB kinase-ε (TBK1/IKKε) complex. The kinase complex activates Interferon regulatory factors 3 and 7 (IRF3 and IRF7), as well as nuclear factor-κB (NF-κB), which induce the transcription of IFN-β and pro-inflammatory cytokines. IFN signal through JAK-STAT to transcriptionally upregulated hundreds of antiviral ISGs. Figure generated with biorender.com.

DDX helicases play important roles in canonical RNA metabolism and cellular homeostasis and are essential for the development and responses to stimuli including infections (84). DExD helicases are found in archaea, prokaryotes, eukaryotes, plants, and even some viruses that encode DExD/helicases (85). For example, flaviviruses encode for NS3 viral helicase, which has similarly conserved motifs I-VI to coordinate ATP hydrolysis and substrate binding (Fig. 2A)(86). Since all of these RNA helicases encode a conserved catalytic core (Fig. 2A), it suggests that the family was expanded from a common ancestor (87). Indeed, orthologous genes in the DExD family were formed by duplication of ancient genes followed by divergent evolution and many have equivalent functions (87, 88). For instance, numerous DDXs in various species participate in ribosome biogenesis: in *E.coli*, four out of the five helicases, in yeast, 14 out of 25 DDX and in humans, at least 18 DDXs contribute to this process (89–92). However, outside the helicase core, helicases have evolved additional domains to expand their function. For example, the *T. thermophilus* protein Hera utilizes an RNA recognition motif to target specific RNA, and the *E.coli* DbpA employs a C-terminal ancillary domain to identify a particular hairpin within the 23S rRNA (80, 93, 94). These auxiliary regions can also modulate the biochemical properties of individual helicases. For instance, the N-terminal extension of the human DDX19 negatively regulates its ATPase activity (95). In humans, paralog pairs like DDX43 and DDX53 feature a KH motif, DDX21, and DDX50 contain the GUCT domain. Conversely, DDX41 has a Zn motif and DDX58 has an N-terminal caspase activation and recruitment domain (CARD)(96, 97, 98). These regions outside of the helicase core also aid in substrate recognition. Although the significance of N- and C-terminal domains in most human DDXs remains unclear, these distinct domains likely play vital roles in helicase localization within specific cellular compartments, interaction with binding partners, and targeting to specific RNAs substrates, thereby imparting functional specificity (59, 77–83, 99).

## PARALOGOUS DDX PROTEINS: FUNCTIONAL REDUNDANCY AND DIVERGENCE

In higher organisms, more DDX paralogs are present, partly due to gene duplication and neofunctionalization of the paralogs (100). Gene duplication primarily drives genome expansion, often in response to various selection pressures. The implication is that the duplicate genes are preserved in the genome because they display, at least in part different functionality, providing a survival advantage (101). However, how paralogs transition from initial redundancy to neofunctionalization is still unclear. While many paralogs overlap in function, potentially to buffer against mutations, they also have distinct functions and higher organisms have a higher number of paralogs (96). For example, human DDXs exhibit established paralog relationships such as DDX2A/B, DDX19A/B, DDX3X/Y, DDX39A/B, DDX5-DDX17, DDX43-DDX53, DDX10-DDX18, DDX47-DDX49, and DDX21-DDX50 (Fig. 2B) (102). Despite a limited understanding of many DDX paralogs’ functions, we will explore some paralog relationships.

DDX3 is an RNA helicase that is encoded by X-linked and Y-linked paralogs *DDX3X* and *DDX3Y*, which have orthologous proteins in a wide range of eukaryotes. However, there is only one paralog in yeast Ded1p, Xenopus An3, mouse PL10, and *Drosophila* Belle. While DDX3X and DDX3Y are 92% identical, the amino acid differences primarily occur in the N-terminus in the intrinsically disordered domains, outside of conserved motifs (103). Their expression patterns vary; DDX3X is localized on the X-chromosome and DDX3Y is localized on the Y-chromosome, and are thus subject to sex-specific epigenetic regulation (104).

DDX3X has known roles in RNA transcription, splicing, stress granule formation, export, translation, and cell cycle regulation and is broadly expressed in somatic tissues. By contrast, DDX3Y has a lower and less consistent expression and regulates spermatogenesis (104–108). DDX3X influences innate immunity, cancer (108, 109), and viral infection, aiding in the replication of viruses such as herpes simplex virus, HCV, influenza A (IAV), Japanese encephalitis virus (JEV), human immunodeficiency virus (HIV), and arenavirus (110–115). The proviral mechanisms vary depending on the virus; for example, it helps export unsliced or partially spliced HIV-1 transcripts by cooperating with the canonical CRM-1 exporter and promotes viral mRNA translation by interacting with the HIV Tat (112, 116). DDX3X promotes JEV translation by interacting with both 5′ and 3′ structured untranslated regions (UTRs) (114). DDX3X also stimulates arenavirus replication by both negatively regulating interferon and by interacting with viral protein to aid in viral RNA replication (111). DDX3X also displays antiviral roles; DDX3X stimulates interferon production, interacts with hepatitis B nucleocapsid to inhibit reverse transcription or downregulate autophagy, and promotes stress granules-mediate interferon activation during IAV infection (117–122). Despite its complex roles in viral infections, antiviral inhibitors targeting the helicase activity of DDX3X have been developed (123, 124). Comparatively, less is known about DDX3Y, but emerging research suggests it shares redundant functions with DDX3X in protein synthesis by facilitating cap-dependent and cap-independent translation through interaction with eIF4F and eIF3 (125). Similarly, in cancer, ectopic expression of DDX3Y restores tumor growth when DDX3X is mutated (105, 126). However, mechanistic studies found that DDX3X has weaker ATPase activity than DDX3Y. Many RBPs, especially those with intrinsically disordered regions, concentrate specific proteins and exclude others within phase-separated, membrane-less compartments, affecting cellular and viral metabolism (127). While both paralogs can form such condensates, DDX3Y promotes liquid-liquid phase separation more strongly (128). However, DDX3Y promotes the aggregation of pathogenic proteins such as TDP-43, potentially explaining sex-specific differences in the incidence of amyotrophic lateral sclerosis (128). In addition, DDX3X and DDX3Y boost interferon expression in cell culture but display incomplete redundancy in mice during bacterial infection (129). It is likely that evolutionarily, these homologs were both retained primarily due to sex-specific differences. For instance, females have missense mutations and males have truncating mutations in DDX3X, with compensations occurring due to the presence or absence of the DDX3Y paralog (103). These initial studies suggest that DDX3 paralogs may share redundant functions but also have distinct biochemical and host defense activities. Given that DDX3X and DDX3Y share high sequence homology, a better understanding of the shared and distinct functions of these paralogs will be important for the design of therapeutics, and for understanding sex-biased diseases.

## DDX39A AND DDX39B: SPECIALIZED ROLES IN RNA METABOLISM AND CELLULAR STRESS RESPONSE

Another pair of well-studied paralogs are DDX39A and DDX39B, which have 89% sequence identity and play well-defined roles in mRNA export (130). While there is only one ortholog in *Drosophila* (*Hel25E*) and yeast (*Sub2p*), there are two paralogs in fish and mammals, suggesting a relatively recent gene duplication event (131, 132). DDX39A and DDX39B also have roles in RNA export; functionally, the *Drosophila* Hel25E, the yeast Sub2, and the *C. elegans* UAP56 are essential for mRNA export in those species, emphasizing the importance of this helicase (133–135).

However, individual helicases are not essential in humans (136, 137), and, either DDX39A or DDX39B can compensate for the loss of the yeast Sub2 homolog, suggesting functional overlap (131, 138). Both paralogs are part of the human TREX mRNA complex and interact with the ALY/REF adapter protein that engages with the NXF1 nuclear receptor (139, 140). DDX39A interacts with CIP29, while DDX39B does not and each paralog binds different mRNAs for export (139, 141). In addition, DDX39A and DDX39B export circRNAs of distinct lengths (142) indicating that differences in exported cargo extend to other substrates besides mRNAs. While both paralogs have *in vitro* helicase activity and can unwind short duplexes, some differences exist. For instance, DDX39B unwinds RNA-DNA hybrids better than dsRNA, and the activity of DDX39A is stimulated by its binding partner CIP29 (58, 59). In addition to its canonical role in RNA export, DDX39B but not DDX39A is involved in splicing regulation by unwinding the U4/U6 snRNA duplex (143–146). Both paralogs are implicated in some cancers but have distinct mechanisms (147–151). For example, DDX39B but not DDX39A stabilizes *BRCA1* mRNA in breast cancer and enhances the splicing/export of the *CDK6/CCND1* and *FUT3* oncogenic genes in colorectal cancer (136, 148, 152). Conversely, DDX39A is oncogenic in lung squamous-cell carcinoma, bladder cancer, mesothelioma, and hepatocellular carcinoma (149, 151, 153, 154). Other less characterized functions for DDX39B include inhibition of p65 phosphorylation, leading to NFkB signaling downregulation (155). DDX39A promotes VSV infection by retaining TRAF3/6 and the mitochondrial antiviral-signaling protein (MAVS) in the nucleus of HEK293T cells and blocking their antiviral activities (72, 155). In addition, DDX39B promotes the export of IAV RNA segments and interacts with various influenza proteins to enhance viral replication (156–159). On the other hand, DDX39A but not DDX39B blocks chikungunya virus infection (137). While these studies highlight that DDX39A and DDX39B share some cellular roles but also exhibit distinct functions. However, the underlying mechanisms that drive these phenotypic differences are not well understood. Both DDX39A and DDX39B are expressed ubiquitously in a majority of human tissues, and although their levels are similar in proliferating cultured cells DDX39A is subject to cell cycle regulation (131). Like other DDXs, DDX39A and DDX39B differ mainly at the N-terminus, leading us to propose that interactions with specific proteins and distinct PTMs drive differences in function (141).

The presence of DDX RNA helicases in all kingdoms of life and their expansion in eukaryotes indicate that DDXs play indispensable roles in RNA metabolism. In the next section, we will describe the essential roles that DDXs play in innate immunity.

## CANONICAL RNA HELICASES IN INNATE IMMUNITY

Innate immunity is characterized by self-non-self recognition. The initial step of defense against invading pathogens requires the recognition of foreign pathogen-associated molecular patterns (PAMPs) by pattern recognition receptors (PRRs) many of which are nucleic acid-binding proteins. For RNA viruses, RNA-binding proteins including helicases bind features associated with viral RNAs (Fig. 2). All RNA viruses produce dsRNAs and many produce RNAs with 5′ triphosphates, and as such there are diverse RBPs that recognize these features. Canonical RIG-I-like receptors (RLR), retinoic acid-inducible gene I (RIG-I/DDX58), melanoma differentiation-associated protein 5 (MDA-5/IFI1), and laboratory of genetics and physiology 2 (LGP2/DHX58), which are part of the DExD/H-box family of helicases are cytoplasmic sensors of viral RNA (160). RIG-I preferentially recognizes short double-stranded RNAs (dsRNA) with 5′triphsophates on uncapped viral genomes, while MDA-5 predominantly recognizes long stretches of dsRNA (161). In addition, RIG-I can recognize 5′-diphosphate moiety on the genome of reovirus (162). In addition to binding these features, RIG-I can also bind the uridine-rich region of the HCV 3′ UTR and sequences within the RNA of the N-gene in the genome of the Hantaan virus (163, 164). The role of LGP2 in innate immunity is less well characterized; LGP2 can bind viral dsRNAs (160), potentially aiding MDA-5-mediated viral sensing (165).

In addition to cytosolic sensing of positive sense RNA viruses which replicate in the cytoplasm, RLRs can also sense DNA viruses, some bacteria, and negative sense RNA viruses such as IAV. In the latter case, RIG-I relocalizes from the cytoplasm to the nucleus and recognizes a panhandle structure in the IAV genome (166, 167). DNA viruses such as herpes simplex virus (HSV) (168) and Kaposi′s sarcoma-associated herpes virus (KSHV) can also activate RIG-I-mediated signaling through DNA-dependent RNA polymerase III and the production of 5′ triphosphate RNAs from their DNA genomes (169–171). Furthermore, RIG-I can also recognize RNA secreted from pathogenic intracellular bacteria such as *Listeria monocytogenes* (172, 173) and *Mycobacterium tuberculosis* (174). Therefore, RLRs sense a wide variety of pathogens to activate host defense mechanisms.

In addition to the helicase core, RIG-I and MDA-5 have two CARD domains. Upon recognition of foreign features on viral RNAs through the helicase core and their C-terminal domains, RLRs trigger an ATP-dependent conformational change in the CARD domains that allow for oligomerization and interaction with the MAVS adaptor in the mitochondria (175). In addition, posttranslational modifications on the RLRs can activate or inhibit activation including phosphorylation, ubiquitylation, SUMOylation, ISGylation, and acetylation (73, 176, 177). Activated MAVS leads to TBK1 and IKKε mediated phosphorylation of the IRF3/7 transcription factors and activation of NF-κB, leading to the expression of interferons (IFNs) and pro-inflammatory cytokines (178, 179). IFN signal in an autocrine and paracrine manner and bind to their receptors thus activating the JAK/STAT signaling pathway, which leads to the induction of hundreds of IFN-stimulated genes (ISGs), resulting in a strong antiviral response (179). While RLRs can be constitutively expressed, they are also SGs, which transiently amplify RLR-dependent innate activation.

## ADDITIONAL RNA HELICASES INVOLVED IN RLR-MEATED IFN ACTIVATION

**Fig 3:**
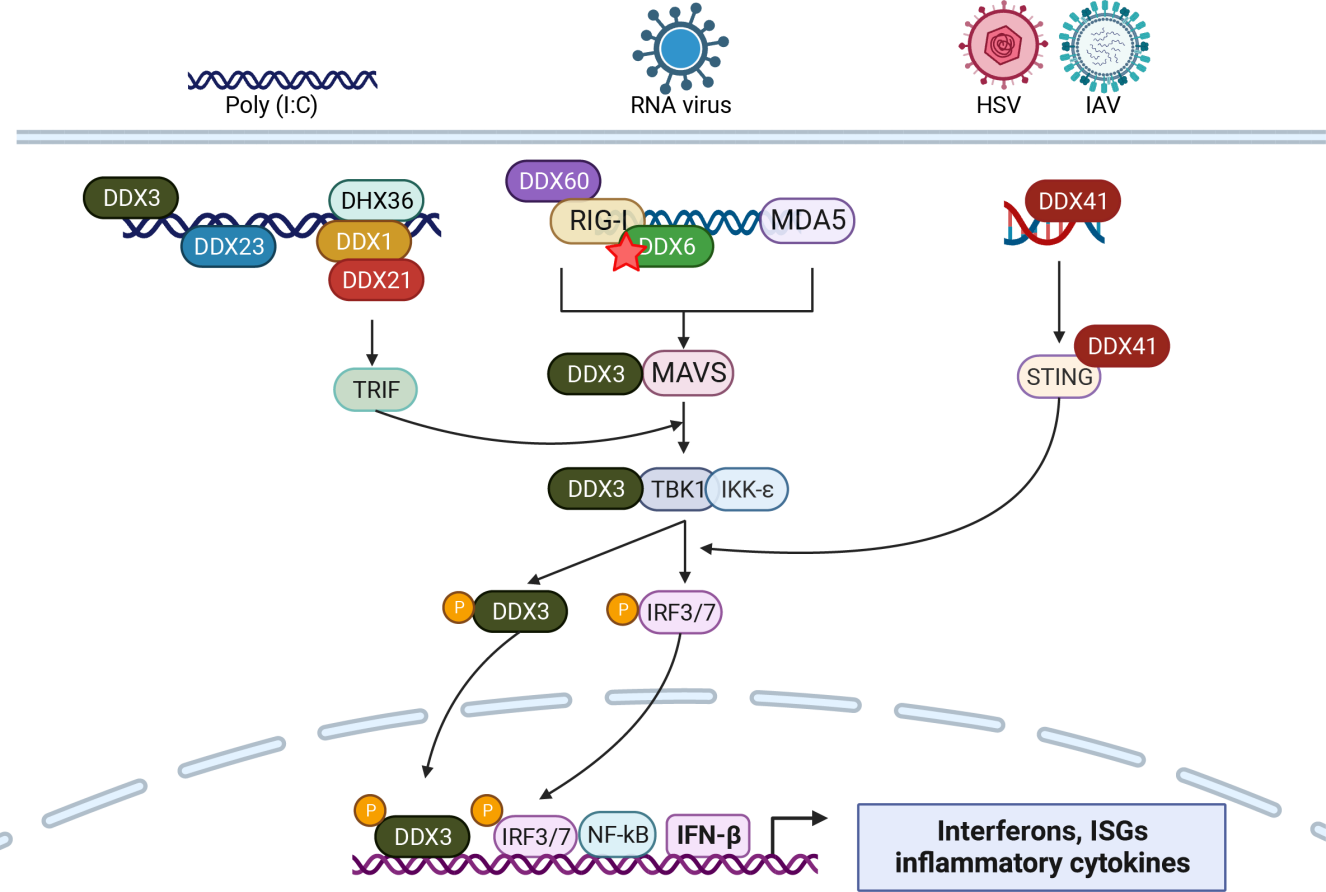
RNA helicases sense viral RNA and regulate IFN activation. DDX3X recognizes viral RNA and interacts with MAVS and TBK1, which phosphorylates IRF3/7 and DDX3; phosphorylated-DDX3 indirectly activates IFN-β transcription to enhance IFN production. The DDX1/DDX21/DHX36 complex binds to poly (I:C) dsRNA and activates IFN through the TRIF adaptor. DDX41 senses viral DNA and cytosolic DNA produced in response to IAV infection and interacts with the Stimulator of interferon genes (STING), which enhances IFN production. DDX6 and DDX60 enhance RIG-I-mediated viral sensing. DDX23 interacts with the poly (I:C) dsRNA and stimulates the innate immune system *via* TRIF. Figure generated with biorender.com.

In addition to the canonical RLRs, multiple DDX helicases either act as additional viral sensors or as regulators of RLR-mediated IFN activation (Fig. 3). DDX3X acts at multiple steps of RLR signaling: can bind abortive HIV transcripts, potentially by binding to a hairpin structure at the 5′ m7GTP cap moiety (107, 118) or by interacting with MAVS (180) to stimulate downstream signaling (180) as well as the IKKε, IKKα, and TBK1 kinases (181–183). There is feedback too, as TBK1 can phosphorylate DDX3X, which then goes to the nucleus and stimulates interferon transcription by helping recruit IRF3 and transcriptional co-activator p/300 to the IFN-β promoter (183, 184). During IAV infection, DDX6 binds to viral RNA and recruits it to RIG-I to enhance RLR signaling (185). Similarly, DDX60 is an accessory factor that enhances the interaction between RIG-I and dsRNA (186, 187). DDX1, DDX21, and DHX36 form a complex that binds viral dsRNAs and induces IFN signaling through the TRIF adapter (188, 189). DDX41, which senses cytoplasmic viral DNA from HSV or mitochondrial DNA released during IAV infection to activate STING (190, 191), and DDX23, which detects viral dsRNAs and binds to TRIF upon stimulation with poly (I:C) or infection with VSV (192). These examples thus illustrate that many RNA helicases sense PAMPs to activate innate immune defense mechanisms.

## FUNCTIONAL INTERPLAY BETWEEN HOST RNA HELICASES AND GENOMES OF RNA VIRUSES

Viruses encode structural elements to promote replication. Some of these structures are sensed by the helicases described above to induce IFN signaling in mammals. However, successful viruses evade or subvert the antiviral IFN pathway (193–195). In addition, many viruses replicate in arthropods and other organisms that lack an IFN pathway. DDX recognition of viral RNAs can impact viral replication independently of IFN signaling and thus plays a key role in antiviral immunity across evolution and in the face of viral antagonism of IFNs

**Fig 4:**
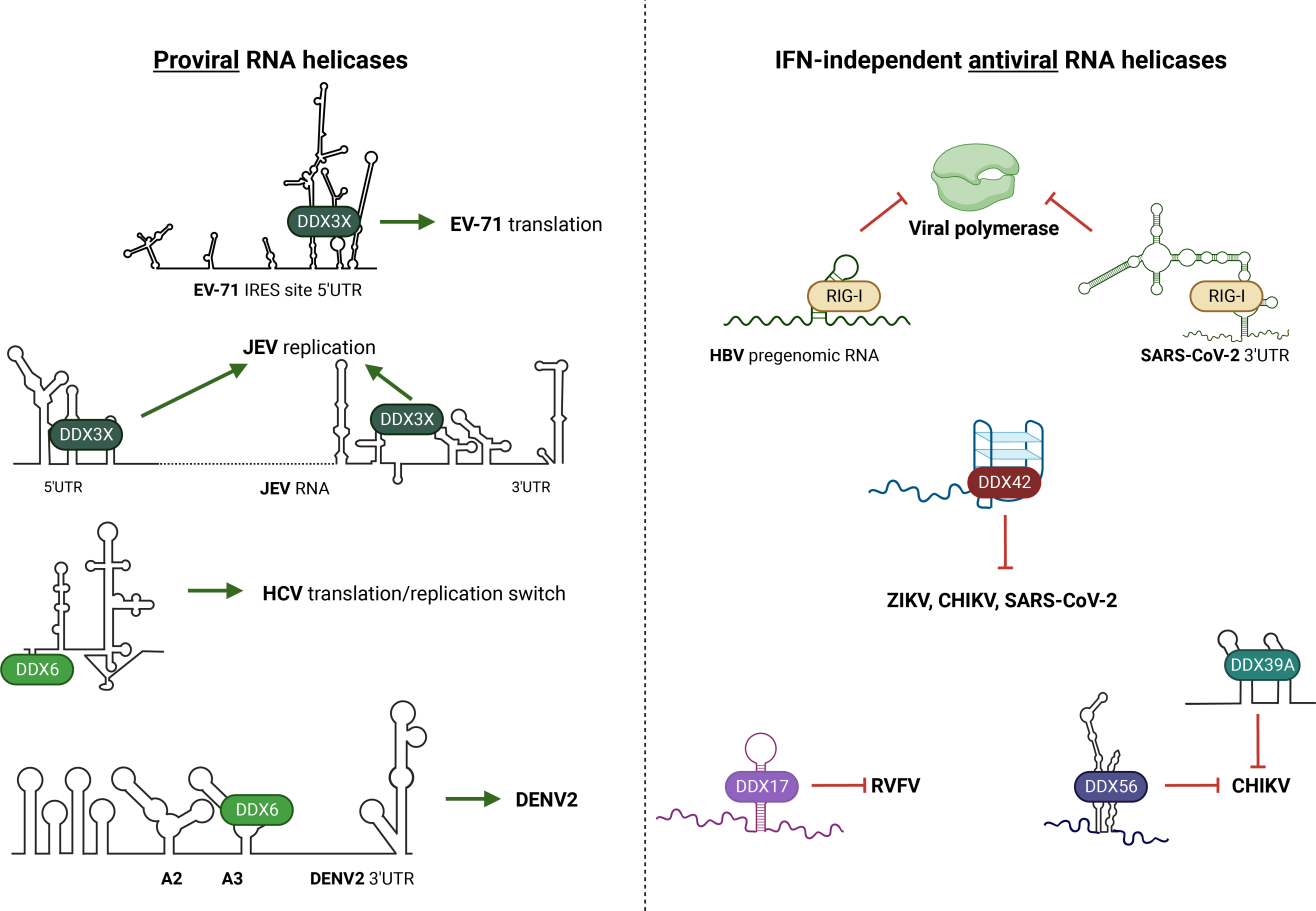
Noncanonical mechanisms of proviral and antiviral helicases through RNA viral structures. (Left) Proviral DDX helicases. DDX3X binds to the EV-71 IRES site and upregulates viral translation (198). DDX3X binds to the 5′ and 3′ UTR and promotes JEV replication (114). DDX6 binds to the 5′UTR of HCV to modulate the interaction of miR-22 with the genome and promote the switch between translation and replication (199, 200). DDX6 specifically binds to the A3 structure on DENV-2 3′ UTR promoting the DENV2 replication (201). (Right) IFN-independent antiviral RNA helicases. RIG-I (DDX58) binds to the 5′-ε stem loop on the pregenomic RNA of HBV and inhibits the viral polymerase (168). RIG-I binds to the 3′ UTR of the SARS-CoV-2 genome and inhibits the viral replicase in an ATPase-independent manner (196). DDX42 binds to viral RNA and restricts replication potentially by unwinding G4 structures (202). DDX17 binds to secondary RNA structures on the RVFV genome to control replication (203). DDX56 restricts alphavirus replication by binding to stem-loop viral RNAs and promoting RNA decay (204). DDX39A binds to the conserved sequence element of CHIKV and restricts viral replication (137). Schematics created using data in cited papers. The figure was generated with biorender.com.

While RIG-I is classically known to bind viral RNAs to induce IFNs, RIG-I also has IFN-independent activities including occlusion of the viral polymerase (168, 196, 197) During gammaherpesvirus infection, RIG-I inhibits the nuclear translocation of the replication and transcription activator and other viral proteins to repress viral transcription (197). For hepatitis B virus (HBV), RIG-I directly interacts with viral RNAs and restricts replication independently of RLR signaling activation (168, 196). Specifically, RIG-I recognizes the 5′-ε stem loop of the pregenomic HBV RNA (Fig. 4) and prevents the viral polymerase from binding and replicating its genome (168). During SARS-CoV-2 infection, instead of binding through the helicase core, RIG-I binds the 3′ UTR of the viral genome through the C-terminus and restricts viral replication in an ATPase-independent manner (196). In contrast to canonical triphosphate sensing, C-terminal bound RIG-I does not undergo conformational change and cannot interact with MAVS to activate the IFN pathway. Rather, RIG-I binding to the SARS-CoV-2 3′ UTR sequesters it away from the viral RNA-dependent RNA polymerase (196).

DDX42 is involved in spliceosome assembly (205, 206). In addition, DDX42 was identified as a G-quadruplex(G4)-binding protein (207), which are proteins that bind to guanine-rich secondary nucleic acid structures that form stacks and are found in both cellular and viral RNAs (208–210). Recently, DDX42 was found to bind to the genomes of several positive-sense RNA viruses including ZIKV, CHIKV, and SARS-CoV-2, and block replication (202). However, DDX42 did not impact negative strand viruses such as vesicular stomatitis virus, influenza, or measles virus suggesting viral specificity. In addition, DDX42 does not modulate ISG induction suggesting that the antiviral activity of this helicase is independent of IFN signaling. While not experimentally shown, the authors hypothesize that DDX42 binds a predicted G4 structure found in DDX42-sensitive RNA-positive viruses to block viral replication (Fig. 4).

DDX17 and the paralog DDX5 can form a heterodimer (211) as well as act independently and these proteins impact transcription, RNA splicing, processing, and degradation (212–216). Interestingly, in the context of viral infection, these paralogs play distinct roles. DDX17 but not DDX5 is a positive regulator of HIV-1 infection by enhancing viral RNA packaging and efficient splicing of HIV-1 transcripts (217). Similarly, DDX17 but not DDX5 controls infection of bunyaviruses such as Rift Valley fever virus (RVFV), without affecting ISG expression (203). Furthermore, DDX17 binds to a specific stem loop on the RVFV genome (Fig. 4), suggesting that DDX17 has a direct antiviral function by interacting with viral RNA (203).

DDX56 is an RNA helicase with known roles in ribosome biogenesis through RNA binding and remodeling (218, 219). DDX56 has been implicated in both promoting and restricting infection (220, 221). Proviral activities are largely mediated through interactions of DDX56 with cellular or viral proteins, while antiviral activities are driven through direct interactions with viral RNAs. For example, DDX56 acts as a negative regulator of RLR signaling by interacting with IRF3 to compete with importin-β3 for export (222) thus antagonizing interferon activation after poly (I:C) and Sendai virus stimulation. DDX56 also binds to the WNV capsid protein and promotes the assembly of infectious viral particles without impacting RNA replication (223, 224). DDX56 interacts with IAV NS1 to enhance infection (225). In contrast to functions driven by protein-protein interactions, DDX56 binding to a stem loop encoded in the CHIKV genome attenuates infection by destabilizing the viral genomic RNA in an interferon-independent manner (Fig. 4)(204).

DDX39A also play an important role in viral infections. DDX39A promotes infection of VSV by unknown mechanisms (72, 155). DDX39A also has antiviral functions independent of interferon signaling by binding the genome of alphaviruses to restrict infection (137). To accomplish this, DDX39A relocalizes from the nucleus to the cytoplasm and associates with ALY/REF to bind a stem loop that is the most conserved structure across alphaviruses (204). DDX39A binding to the CHIKV genome reduced replication of the negative sense antigenome and dsRNA intermediates thus blocking replication (137).

DDX24 and DDX49 also interact with viral RNA to control infections. DDX24 is a predominantly nucleolar protein involved in ribosomal processing. DDX24 negatively regulates interferon signaling by sequestering dsRNA away from RLRs, and by impeding the recruitment of IRF7 to the signaling complex (74, 226). DDX49 is a nuclear protein that impacts RNA transcription, stability, and export (63). However, during infection with Kaposi’s sarcoma-associated herpesvirus (KSHV), DDX24 and DDX49 bind to multiple KSHV mRNAs and reduce viral reactivation (227). While the exact mechanism is not known, the authors suggest that DDX24 and DDX49 binding to KSHV mRNAs induces IFN production.

## VIRAL HIJACKING OF RNA HELICASES FOR REPLICATION

While most viruses encode their helicases, many also usurp cellular DDX helicases to enhance viral gene expression, translation, and packaging (228, 229). Furthermore, viruses can antagonize DDXs, preventing them from performing their primary cellular homeostatic role.

DDX3X has diverse roles in RNA biology including well-studied roles in translation (Fig. 3). Many viruses utilize cap-independent translation through internal ribosome entry sites (IRES) and DDX3X facilitates the translation of these highly structured IRES elements. Clear roles have been established with JEV, a neurotropic flavivirus, DDX3X interacts with the NS3 and NS5 viral proteins and with the highly structured 5′ and 3′ UTRs; these interactions promote JEV translation and replication (114). In the case of the positive strand enterovirus 71 (EV71), which is responsible for hand-foot-mouth disease, DDX3X is recruited to the IRES to promote viral translation through its helicase activity (198). Furthermore, the IRES-promoting activity of DDX3X is no longer important when a specific secondary RNA structure is destabilized suggesting specific recruitment of the helicase to the viral RNA structure (198). These studies indicate that different viruses recruit DDX3X to promote viral translation.

DDX6 also plays diverse cellular roles in translational repression, mRNA, decapping, and miRNA repression (230, 231). DDX6 enhances viral replication of diverse Flaviviridae. For HCV, DDX6 promotes viral RNA replication dependent on its helicase activity (199, 200). Specifically, DDX6 binds to the structured 5′ UTR of HCV and facilitates the transition from viral translation to viral replication by modulating the interaction of a miRNA with a specific binding site in the 5′UTR (199). In the case of DENV, DDX6 preferentially binds to the A3 dumbbell within pseudoknot RNA structures (A2 and A3 dumbbells) in the 3′ UTR and facilitates viral RNA replication (66, 201). *In vitro* assays further showed that when binding to its preferred A3 RNA substrate in DENV RNA, DDX6 exhibits an enhanced ATPase activity compared to low basal ATPase activity in the presence of tRNA or the other A2 dumbbell. The hypothesis is that the interaction of the DDX with a preferred substrate or with a preferred binding partner can alter the positioning of the two RecA domains resulting in an increased ATPase activity (65). These results indicate that DDX6 recruitment to specific viral structures promotes infection and this also reduces the ability of DDX6 to enhance RLR-dependent IFN signaling (185).

Additional helicases that interact with viral genomes and enhance viral replication include DDX1 with the 3′ UTR (+) of HCV (232), and DDX5 with the 3′ UTR of JEV (233). However, functional and structural requirements between many of such interactions are not well understood. Altogether it is clear that specific DDX RNA helicases bind viral genomes and alter specific structures or local interactions to enhance viral replication.

## CONCLUDING REMARKS

DDX helicases, a conserved group of RNA-binding proteins, regulate various cellular RNA processes, including ribosome biogenesis, translation, and splicing, which require recognition and processing of RNA structures (6, 11, 35, 60). Emerging research suggests their involvement in innate immunity by interacting with viral RNA structures and other proteins implicated in these signaling pathways. Importantly, the binding of these helicases to RNA structures encoded by viral RNAs can impact infection through interferon-dependent and interferon-independent mechanisms. While most DDXs lack sequence-specific RNA-binding motifs their functional specificity argues for specific modes of interaction and recognition of RNA structures (81, 234). Several studies reveal complex regulation of basal *in vitro* enzymatic activities through substrate recognition and interaction with protein-binding partners (58, 59, 62, 64, 66). However, there is an incomplete understanding of how sequence and structural differences among helicases impact their enzymatic activities and substrate selection. Current studies suggest that protein-protein interactions and PTM, particularly within the divergent domains, may be key to functional specificity. In the context of viral infections, several interactions between DDX helicases and viral genomes have been identified, but the specific structures being recognized remain poorly characterized. Identifying viral RNA features bound by RNA helicases either alone or in concert with other RNA-binding proteins will provide additional insight into determinants of RNA substrate selection and functional specificity. This understanding is vital before the development of specific DDX host-directed antivirals. Moreover, gaining a deeper insight into the regulation of DDX RNA helicases, alongside the increasing availability of viral RNA structures will advance our understanding of host-pathogen interactions in the context of an ongoing arms race between viruses and their hosts.

## SUPPLEMENTAL MATERIAL

Table S1 jvi.00040-24-s0001.xlsx DDX UniProt protein IDs used for generating the phylogeny tree.

ASM does not own the copyrights to Supplemental Material that may be linked to, or accessed through, an article. The authors have granted ASM a non-exclusive, world-wide license to publish the Supplemental Material files. Please contact the corresponding author directly for reuse.
